# *Allanblackia* Oil: Phytochemistry and Use as a Functional Food

**DOI:** 10.3390/ijms160922333

**Published:** 2015-09-15

**Authors:** Sara L. Crockett

**Affiliations:** Department of Pharmacognosy, Institute of Pharmaceutical Sciences, Universitaetsplatz 4/I, University of Graz, Graz 8010, Austria; E-Mail: sara.crockett@uni-graz.at; Tel.: +43-316-380-5525; Fax: +43-316-380-9860

**Keywords:** *Allanblackia*, Clusiaceae, oil, seed, functional food, saturated fatty acid, stearic acid, oleic acid

## Abstract

The consumption and commercial exploitation of *Allanblackia* (Clusiaceae) seed oils is of current interest. The favorable physicochemical characteristics of *Allanblackia* oil (solid at room temperature; high stearic acid content) lend food products that contain it (*i.e.*, vegetable-based dairy products, ice cream, spreads) health advantages over others that contain higher levels of lauric, myristic, and/or palmitic acids, which can increase blood cholesterol levels. Such considerations are important for individuals prone to cardiovascular disease or with hypercholesterolemia. Domestication projects of several *Allanblackia* species in tropical Africa are underway, but wildcrafting of fruits to meet the seed demand still occurs. Proper species authentication is important, since only authenticated oil can be deemed safe for human consumption. The chemical constituency of *Allanblackia* seed oils, and potential roles of these phytochemicals in preventive strategies (e.g., as part of a healthy diet) and as pharmacological agents used to treat chronic disease were examined in this review. The primary and secondary metabolite constituency of the seed oils of nearly all *Allanblackia* species is still poorly known. The presence, identity, and quantity of potentially bioactive secondary metabolites in the seed oils, and pharmacological testing of isolated compounds were identified as important directions for future research.

## 1. Introduction

On a worldwide scale, the supplies and consumption of oils and fats have generally been described in terms of 17 commodity oils, four that originate from animals and thirteen that are derived from plants, namely soybean, palm, rape/canola, sunflower, coconut, palmkernel, cottonseed, groundnut (peanut), olive, corn, sesame, rice bran, and flaxseed oil. During recent decades, as information about the negative health effects of animal fat consumption has accumulated, higher consumption rates of fats and oils from plants have been documented. In addition, the demand for oils and fats from alternative plant sources has steadily increased, driven by several factors including: the demand for food from a steadily growing population with more financial resources, demand for biodiesel (food-fuel debate), price increase of some oils due to the rising costs of agricultural production, storage, and transport, fluctuations in oilseed yield due to poor climatic conditions in many parts of the world, and speculation [1]. In particular, due to the fact that more than 90% of the world’s biodiesel is currently produced from edible vegetable oils, significant research efforts are being invested in the discovery of alternative plant-based oil (both edible and non-edible) sources [2].

At the same time, an ever increasing number of consumers believe that foods contribute directly to their health and this has, in turn, driven food producers and manufacturers to produce foods that not only satisfy hunger and provide valuable nutrients, but can be used as preventative medicine to improve both physical and mental health [3]. The World Health Organization (WHO) and the Food and Agriculture Organization of the United Nations (FAO) have described diets and lifestyle habits that can contribute to the development of such chronic diseases as cancer, osteoporosis, coronary heart disease, obesity, periodontal disease, and type 2 diabetes [4]. Therefore, changes in diet and potentially the inclusion of functional foods could lead to the prevention of chronic diseases. As a growing market, functional foods have steadily increasing economic importance. With regard to plant-based oils as functional foods, information on both the primary (*i.e.*, triacylglycerols and fatty acids) and secondary (e.g., phosopholipids, sterols, tocopherols, carotenoids, phenolics) metabolites in these oils is of interest.

While numerous reviews about bioactive components in the common commodity oils are available, the literature describing primary and secondary constituents in new, alternative, edible plant-based oils is still sparse. Additionally, few studies have specifically focused on the roles of these phytochemicals in preventive strategies (e.g., associated with increased intake as part of a healthy diet) or as pharmacological agents used in the treatment of chronic disease. The current review summarizes information known about the oils expressed from seeds of species of *Allanblackia* Oliv. ex. Bentham (Clusiaceae), a genus that is currently the focus of a high degree of attention with regard to its consumption and commercial exploitation.

## 2. Results and Discussion

### 2.1. Botanical Description and Geographic Distribution

The genus *Allanblackia* is a member of the flowering plant family Clusiaceae Lindley (also Guttiferae Juss. *nom. alt. et cons.*, order Malpighiales), which includes 14 genera and nearly 600 species of trees or shrubs that are primarily distributed in tropical regions of the world. The family comprises the subfamilies Clusieae Choisy (*Clusia* L., *Chrysochlamys* Poepp., *Dystovomita* (Engler) D’Arcy, *Tovomita* Aublet, *Tovomitopsis* Planchon & Triana), Symphonieae (*Lorostemon* Ducke, *Montrouziera* Planchon & Triana, *Moronobea* Aublet, *Pentadesma* Sabine, *Platonia* Martius, *Symphonia* L., *Thysanostemon* Maguire), and Garcinieae Choisy (*Allanblackia*, *Garcinia* L.). Species belonging to the Garcinieae are dioecious and share several morphological characters including the possession of colleters (clusters of mucilaginous secretory hairs), capitate stigmas, frequently non-scaly buds and anthers that open toward the gynoecium, as well as fruits that are indehiscent and baccate, whereby the testa and endocarp are at least partially fused [5]. Sweeney [6] and Ruhfel *et al.* [7] have suggested that *Allanblackia* should be combined with *Garcinia* on the basis of the results of multigene phylogenetic analyses, but the former generic name has been thus far been retained. Both *Allanblackia* and *Garcinia* (mangosteen) contain species with oily seeds that are of potential commercial interest, because two component fatty acids (stearic and oleic) together comprise up to 95% of the total fatty acids present in the seed oil [8].

**Figure 1:**
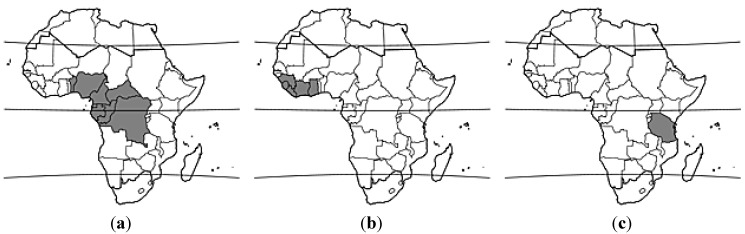
Native geographic distributions of (**a**) *Allanblackia floribunda*; (**b**) *A. parviflora*; and (**c**) *A. stuhlmannii*. Maps reprinted with permission from: PROTA (Plant Resources of Tropical Africa/Ressources végétales de l’Afrique tropicale), Wageningen, The Netherlands. http://www.prota4u.org.

Nine species of *Allanblackia* have been recognized, all of which are restricted in their natural distribution to tropical Africa, according to Bamps *et al.* [9]: *A. gabonensis* (Pellegr.) Bamps, *A. floribunda* Oliv., *A. kimbiliensis* Spirl, *A. kisonghi* Vermoesen, *A. marienii* Staner, *A. parviflora* A.Chevalier, *A. stanerana* Exell & Mendonça, *A. stuhlmannii* Engl., and *A. ulugurensis* Engl. Three of these species (*A. floribunda*, *A. parviflora*, and *A. stuhlmannii*) are of considerable commercial interest (see Figure 1).

**Figure 2:**
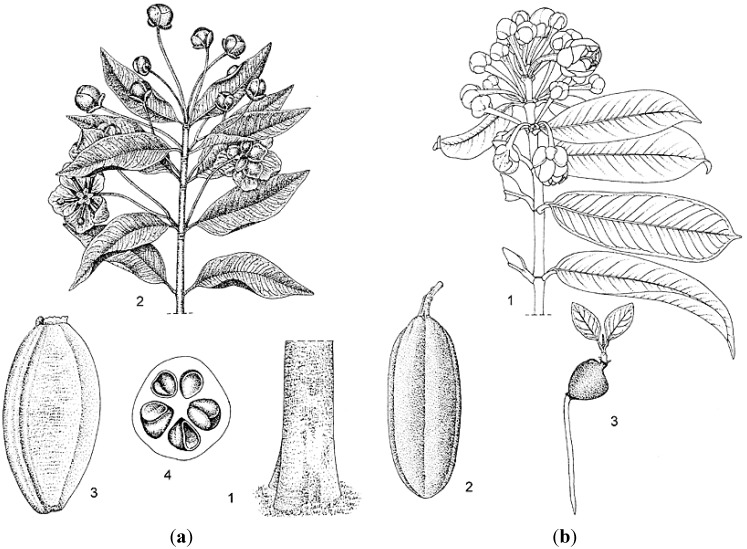
Line drawings detailing characteristic botanical features of (**a**) *Allanblackia floribunda* (**1**, base of bole; **2**, flowering twig; **3**, fruit; and **4**, fruit in cross section showing seeds); and (**b**) *A. parviflora* (**1**, flowering twig; **2**, fruit; and **3**, seedling). Line drawings reprinted with permission from: PROTA (Plant Resources of Tropical Africa/Ressources végétales de l’Afrique tropicale), Wageningen, The Netherlands. http://www.prota4u.org.

*Allanblackia floribunda* (vegetable tallow tree) is a tree species that is commonly distributed in the moist tropical forest zone that extends from Nigeria east to the Central African Republic and the eastern Democratic Republic of the Congo, then south to northern Angola and eastward to Uganda [10]. *Allanblackia parviflora* (also referred to as vegetable tallow tree) has been frequently confused with *A. floribunda* due to their high degree of vegetative morphological similarity. Both are evergreen trees that, when mature, can attain a height of up to 30 m and a trunk diameter of 80 cm; can have reddish-brown bark with small, irregular scales; possess opposite, simple, entire, glabrous, estipulate leaves with short (*ca.* 1 cm) petioles; have unisexual, regular, five-merous, pinkish to reddish flowers; and large, ellipsoid, berry-like fruits with at least 40 and up to 100 seeds (Figure 2). The male flowers of *A. floribunda*, however, have deeply folded disk glands and longer (3–8 cm) pedicels, as compared to male flowers of *A. parviflora*, which have smooth or slightly folded glands and shorter (1–3 cm) pedicels. In addition, the distributional areas of the species rarely overlap in nature, with trees of *A. parviflora* primarily occurring in the forested zone that extends from Guinea and Sierra Leone to Ghana [11].

**Figure 3:**
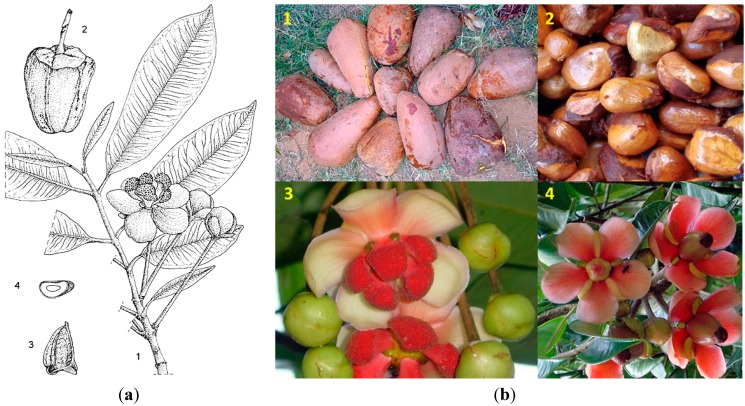
Line drawing detailing characteristic botanical features of (**a**) *Allanblackia stuhlmannii* (**1**, twig with male flowers; **2**, fruit; **3**, seed; and **4**, seed in cross section); and (**b**) photos of *A. stuhlmannii* (**1**, fruits; **2**, seeds; **3**, male flowers; and **4**, female flowers). Line drawings reprinted with permission from: PROTA (Plant Resources of Tropical Africa/Ressources végétales de l’Afrique tropicale), Wageningen, Netherlands. http://www.prota4u.org; photos taken by M. Munjunga reprinted with permission from: ICRAF (World Agroforestry Centre), East & Southern Africa Regional Programme, Dar-es-Salaam, Tanzania. http://www.worldagroforestry.org.

*Allanblackia stuhlmannii* (in Swahili: Mkange), a species endemic to Tanzania, is distributed from Tanga in the North-East Highlands to the Iringa Region in the Southern Highlands, throughout the Eastern Arc Mountains, and occurs in mid-elevation evergreen sub-montane and montane forests. These evergreen trees can attain a mature height of up to 45 m, but have a more slender trunk (*ca.* 65 cm in diameter); dark grey to black bark that is smooth or rarely flaking with square scales; have opposite, simple, entire, glabrous, estipulate leaves with longer (1–2 cm) petioles; unisexual, regular, five-merous, cream to reddish fragrant flowers; and large, oblong to globose or cone-shaped berry-like fruits with at least 60 and up to 140 seeds [12] (Figure 3). See Table 1 for a comparison among the main botanical characteristics of the three species.

Proper botanical identification of the species used as a source of *Allanblackia* seeds is important when considering the use of the seed oil as a functional food or an ingredient in food products. Only when oil derived from the correct plant species and plant part (unadulterated) is used can the product be deemed safe for human consumption and assessed as to whether it has the chemical constituency that it claims. Many data are still lacking for species of *Allanblackia* that could be collected using identification methods such as macroscopic, microscopic, organoleptic, thin-layer chromatography (TLC), high-pressure (HP) TLC, HP liquid chromatography (HPLC) and Fourier transform infrared spectroscopy, all of which are commonly used in the quality control of botanical ingredients. Although domestication projects are ongoing to establish the sustainable cultivation of these species in tropical Africa, the steadily growing demand for *Allanblackia* seed oil has resulted in increased harvesting pressures on wild populations of *Allanblackia* species and, in turn, increased the risk of misidentification and/or adulteration.

**Table 1:** Botanical characteristics of three species of *Allanblackia* of commercial interest [10,11,12].

Species	*A. floribunda*	*A. parviflora*	*A. stuhlmannii*
**Mature height/Trunk Diameter**	30 m/80 cm	25 (–33) m/80 cm	35 (–45) m/65 cm
**Outer bark**	Reddish-brown to blackish; scales small and irregular	Yellowish-brown or reddish-brown; scales small and irregular	Dark grey to black, smooth or rarely flaking with square scales
**Inner bark**	Granular, reddish or brown, exuding clear sap	Reddish-brown with sometimes pale yellow streaks; exuding colorless or pale yellow sap	Red to pale brown with white stripes, fibrous to granular, exuding a clear sap that turns yellowish upon oxidation
**Leaves**	Opposite, simple, entire, glabrous, estipulate; blade elliptical to ovate, rarely obovate; base rounded or cuneate; apex acuminate; 8–25 cm × 3–8 cm; petiole 1 cm	Opposite, simple, entire, glabrous, estipulate; blade elliptical to narrowly obovate; base cuneate; apex acuminate; 12–25 cm × 5–9 cm; petiole 1–1.5 cm.	Opposite, simple, entire, estipulate; blade oblong to elliptical-oblong; base cuneate, apex shortly acuminate; 5–20 cm × 1–7 cm; petiole 1–2 cm
**Inflorescence**	Terminal raceme or panicle with strongly reduced branches or flowers single or in pairs in leaf axils	Terminal raceme or panicle with strongly reduced branches or flowers single or in pairs in leaf axils	Flowers solitary in leaf axils or crowded at the end of branches
**Flowers**	Unisexual, regular, five-merous, pinkish or reddish (rarely white); pedicel 3–8 cm	Unisexual, regular, five-merous, pinkish or reddish, fragrant; pedicel 1–3 cm	Unisexual, regular, five-merous, cream to reddish, fragrant; pedicel (3.5–) 6.5–8 cm
**Sepals**	Orbicular, unequal, outer ones 5–8 mm in diameter, inner ones 12–15 mm in diameter, glabrous	Ovate or obovate, unequal, 6–18 mm × 4–15 mm, glabrous	Orbicular to ovate, unequal, outer ones 4–9 mm in diameter, inner ones *ca.* 2 cm in diameter, pale yellow
**Petals**	Obovate to orbicular, 20–25 mm long, glabrous	Obovate, *ca.* 20 mm long, glabrous	Orbicular to spathulate, 27–37 mm × 18–26 mm, glabrous
**Stamens**	Numerous, in 5 bundles opposite the petals, 10–15 mm long, anthers arranged on the internal face of the bundle; disk star-shaped with deeply folded glands	Numerous, in five bundles opposite the petals, obtriangular, *ca.* 18 mm long, anthers arranged on the internal face of the bundle; disk star-shaped with smooth or slightly folded glands	Numerous, in five bundles opposite the petals, *ca.* 2 cm long, inner surface angled, anthers arranged on the 2 faces of the bundles; disk star-shaped
**Ovary**	Superior, incompletely five-celled, stigma sessile, staminal bundles reduced to a few free 4–5 mm long staminodes, disk glands grooved	Superior, incompletely five-celled, stigma sessile	Superior, incompletely five-celled, stigma sessile; staminal bundles reduced to a few free, *ca.* 4 mm long staminodes
**Fruit**	Large ellipsoid berry 20–50 cm × 5–14 cm, with five longitudinal ridges, 40–80 seeded	Large ellipsoid berry 10–50 cm × *ca.* 15 cm, with five longitudinal ridges, brown warty appearance, 40–100 seeded	Large oblong to globose or cone-shaped berry 16–34 cm × 15–17 cm, 2.5–6 kg, red-brown, 60–140 seeded
**Seeds**	Ovoid, 2.5–3 cm × 1.5–2 cm, enclosed in a pinkish aril; embryo small, embedded in oily endosperm	Ovoid, *ca.* 3 cm × 2 cm, enclosed in a pinkish aril	4-Angular, *ca.* 4 cm × 2–3 cm, one angle with a small fleshy aril; embryo small, embedded in oily endosperm
**Germination**	Seedling with hypogeal germination	Seedling with hypogeal germination; epicotyl 4–5 cm long	Seedling with hypogeal germination

### 2.2. Traditional Allanblackia Seed Oil Use

By the late 19th century, well-established colonial empires such as France, Britain, Belgium and Portugal had already laid claim to large areas of Africa, while developing imperial powers such as Germany and Italy had followed suit on a smaller scale. Colonial powers invested significant efforts in–among other things–the exploration and exploitation of the new colonies as sources of raw materials for European industry [13]. Many alternative oil seeds were identified and studied, in particular by French, Belgian, and Italian institutions conducting research for their colonies in Central Africa, in part to provide the colonies with information about local plants that could be used for food and energy production. During the Great War, *Allanblackia* oil was used as a substitute for cocoa butter during the manufacture of chocolate [14].

The traditional use of the *Allanblackia* seed oil has been reported for *Allanblackia floribunda* (vegetable tallow tree, Kisidwe nuts), *A. stuhlmannii* (kagne butter), *A. ulugurensis* (kagne butter), and *A. parviflora* (vegetable tallow tree) [10,11,12,15] (Proto). Uses for *Allanblackia* oil vary according to the region in which the species occur. In West Africa, in parts of Ghana, Tanzania, Nigeria, and Sierra Leone, the seeds of various *Allanblackia* species (especially *A. parviflora* and *A. floribunda*) occurring in the region have been traditionally collected by local communities for food (e.g., for the production of cooking oil), and more recently, to produce soap [16,17]. After the seeds are ground and pressed to extract the oil, the bitter seedcake can be used as a protein-rich animal feed [18]. The frequency of traditional use of *Allanblackia* oil, however, has decreased over the last 50 years due to the availability of other commercially available oils. The seeds have a substantial value due to the high nutritional value of the oil, as well as its unique physical properties, which are similar in some ways to those of the oil obtained from the seed kernels of the African oil palm (*Elaeis guineensis* Jacq.), but production on a commercial scale had not been attempted until recently [19,20].

After decolonization in Africa, the research efforts to discover new, alternative oilseeds continued. In the 1970s and 1980s, the seed oil from *Allanblackia* fruits harvested in the East Usambara Mountains in Tanzania was exported to Europe in small, but significant quantities [14]. In the 1980s, research efforts were supported by members of the African Safou Network (ASANET), which was initiated in 1980 to conserve indigenous fruit tree genetic resources, and which attempted to establish a market for the seed oil of the Safou or Atanga tree (*Dacryodes edulis*, Burseraceae) [21]. During this time, the researchers observed that some of these alternative seed oils were used for food or integrated into locally-sold cosmetics and/or medicines, and in some cases, regional or international markets existed [22].

The seed oil of *A. parviflora* is being developed as a rural based enterprise in Ghana, Nigeria, Cameroon, and Tanzania [23]. Currently, the “Novella Africa Project”, which involves the corporate entity Unilever PLC (public limited company) and several African research agencies, farmers and NGOs (non-governmental organizations) working towards the development of high-quality consumer products for household use. Through their investments, Unilever PLC has created a stable market for the oil, and this is predicted to expand into a new African industry with a market value exceeding $100 million [24]. In 2007, the European Food Safety Authority deemed *Allanblackia* seed oil (described as oil derived from the seeds of *A. floribunda* and/or *A. stuhlmannii*) as being acceptable for human consumption under specified conditions when included in “yellow fat and cream-based spreads”. The food products that contain *Allanblackia* seed oil include vegetable-based dairy products, ice cream, and spreads [25].

It should be noted, however, that some of the concerns mentioned in Section 2.1 also apply here. The ESFA (European food safety authority) recommended that only species of *Allanblackia* that had been subjected to toxicological studies should serve as sources of raw materials for the seed oil production, but due to the continued prevalence of wildcrafting of materials in some regions and the absence of quality control parameters for many of these species, this recommendation may not always be followed. The samples analyzed by the ESFA were derived from only two species of *Allanblackia* and, thus, did not represent the full species diversity to be found in the tropical rain forests in various geographic regions of Africa.

Results from detailed, systematic, comparative analyses of the chemical composition of seeds from species of *Allanblackia* have not been made publically available. In their application to include *Allanblackia* seed oil in vegetable-based spreads, submitted in 2004, Unilever Deutschland GmbH indicated that some batches of the unrefined seed oil contained benzophenone derivatives (*i.e.*, guttiferone E and F), which could be removed by additional refining processes [26]. The latter compound induces cellular apoptosis and has been reported as a constituent of *A. stuhlmannii* roots [27]. With respect to the use of *Allanblackia* oil as a functional food, therefore, this information serves as evidence that at least the unrefined seed oil contains bioactive secondary metabolites that could exert pharmacological effects. Further *in vitro* and *in vivo* studies are needed, however, to fully investigate the extent of this bioactivity.

### 2.3. Cultivation and Wildcrafting of Allanblackia Seeds

As mentioned above, it is crucial to gather information about the origin of plant-based fats and oils, since both genetic and environmental factors influence aspects of production and trade. *Allanblackia* trees that have been planted for the commercial production of seed oil need significant time to mature, usually seven years or more, before they produce an economic crop, and due to the dioecious nature of the plants, obviously a crop will only be obtained from (productive) female plants. In general, tree crop yields can be affected by seasonal climatic changes, as well as environmental factors such as the use of herbicides, fungicides, or pesticides (e.g., at the moment, only sprayed on seeds prior to planting) and additional advantage is that tree crops grown in tropical Africa can be harvested year-round, although the levels of seasonal qualitative and quantitative variation have yet to be determined for several regions and species [23,28].

The FAO had already identified *Allanblackia* as a crop of high potential interest in 1992, due to the potential for the seed oil (which was listed as a non-edible oil at that time) to become a profitable and sustainable raw material, the development of which could benefit rural communities [29]. Several studies and surveys have been conducted in recent years to assess the socioeconomic progress associated with the establishment of a supply chain for *Allanblackia* oil [20,30,31]. Currently, *Allanblackia* seed (e.g., from *A. floribunda*, *A. parviflora*, *A. stuhlmannii*) is usually collected in the wild or from trees that have been preserved on farm land (trees retained to provide shade for animals and other crop plants, when clearing land for cultivation) for use in cultivation [20,23].

Since the establishment of the “Novella Africa Project” in 2002, significant advances in knowledge about the natural distribution of the most common *Allanblackia* species, as well as their production potential, biology, and ecology have been made. Jamnadass *et al.* [32] and references therein described the coordinated strategies that had been undertaken up until 2010 to domesticate *Allanblackia* trees in such a way as to address both the demands of the market and the varied challenges posed by cultivation and conservation. In addition, more recent studies on seed germination and propagation, sex determination of individuals, population biology, and genetic diversity for selected species in specific geographic locations have been carried out [33,34]. In particular, results of molecular analyses with respect to amplified fragment length polymorphisms (AFLP) have indicated that this method may be useful to differentiate among species, especially when vegetative material only is available for collection in regions where more than one species of *Allanblackia* occur, and that the high levels of AFLP variation suggest useful domestication opportunities [34]. Much data is still lacking, however, for entire species, and significant gaps in the data exist even for the more common species of *Allanblackia* with respect to their reproductive biology, propagation, cultivation, selection, and breeding, which need to be addressed through targeted research.

### 2.4. General Characteristics of the Seed Oil

Like many commonly-used vegetable oils, *Allanblackia* oil consists of well-known triglycerides. Because it contains tocopherol as a minor constituent, it has good storage stability characteristics. Due to its chemical composition and relatively high melting point (*ca.* 34 °C), it can be used to improve the consistency of cocoa butter substitutes, margarine spreads, and other vegetable-based dairy products. The fact that it does not require additional transformation to acquire the desired characteristics is an added advantage. As in the case of other oils, *Allanblackia* oil can be combined with other oils or fats to achieve specific physical properties. When analyzing the saturated fatty acids (SFAs), the high stearic acid content (45%–58% on average) and comparatively low palmitic acid content can be used to chemically distinguish *Allanblackia* oil from palm and palm kernel oils [25].

*Allanblackia* seed oil shares some characteristics with shea butter (from the seeds of *Vitellaria paradoxa* C.F.Gaertn. (Sapotaceae)), making it a valuable and useful raw material in both the food and cosmetic industries. The triglyceride composition of the seed oil indicates that, given the existence of a stable supply chain, *Allanblackia* oil can potentially serve as an alternative in many food and cosmetic products to palm oil, cocoa butter, and shea butter. When considering the implications of the use of *Allanblackia* seed oil as a functional food or functional food component, it is important to keep in mind that individual SFAs have different effects on blood cholesterol levels. Stearic acid is unique in that, unlike other long chain SFAs (>10 carbons), human and animal studies with shea butter and cocoa butter have demonstrated that stearic acid does not alter the levels of total low density lipoprotein (LDL, or “bad” cholesterol) and high density lipoprotein (HDL, or “good” cholesterol) measured in the blood of adults. Other long chain SFAs that often predominate in plant-based oils, including lauric (C12:0), myristic (C14:0), and palmitic (C16:0) acids, lead to increases in blood cholesterol levels and correspondingly increase the risk of the development of cardiovascular disease, unlike stearic acid [35,36,37]. These results indicate that including fats and oils (or functional foods containing these) that are rich in stearic acid, as opposed to other SFAs, in the diet could be advantageous, particularly for hypercholesterolemic individuals [38].

#### 2.4.1. *Allanblackia floribunda* Seed Oil

An early study of *A. floribunda* seed oil reported that the fatty acid constituents were made up of primarily stearic acid (56.8%) and oleic acid (39.4%), followed by minor amounts of palmitic acid (3.2%), linoleic acid (0.4%), and eicosanoic acid (0.2%). Triglyceride components consisted mostly of 2-oleostearin (76.2%), 1-stearo-diolein (15.5%), and 2-oleopalmitostearin (5%) [39]. In a more recent study, in which seeds collected from seven individuals of *A. floribunda* growing in the Congo were extracted via Soxhlet extraction (cyclohexane), a yield of 60%–65% oil (*w*/*w*) was obtained. This seed oil consisted primarily of a saturated fatty acid (stearic acid, 61%–63%) and a monounsaturated fatty acid (oleic acid, 35%–36%) [22]. Another study, in which *A. floribunda* seeds were collected from trees occurring in Cameroon, ground and extracted using a Soxhlet extraction (hexane) technique, revealed that the seeds contained 62.5% oil (*w*/*w*) and 6.7% ash. The oil, examined with gas chromatography, consisted of 62.6% of saturated fatty acids (61.3% of which were stearic acid) and 36.7% of monounsaturated fatty acids (of which 36.6% were oleic acid), as well as 0.7% polyunsaturated fatty acids [40]. Analyses of seeds collected from *A. floribunda* trees growing in Nigeria yielded similar results, demonstrating that the seeds contained on average 60.4% oil, 1.7% ash, 4.1% fiber, and 32.6% carbohydrates. Positive chemical reactions that indicated the presence of minor amounts of alkaloids, flavonoids, saponins, and tannins were observed, although it must be noted that these results must be confirmed by alternative methods (e.g., TLC, HPLC), since false positive test results can occur with the methods described [41].

While few studies have thus far been conducted to examine intra- and interpopulation differences in *Allanblackia* seed oil yield and composition, one such study in which 17–40 fruits were sampled from each of 70 *A. floribunda* trees growing wild at four sites from within the natural range of the species in Cameroon revealed that the fatty acid content of the seeds ranged from 44.2%–66.1% (stearic acid) and 25.0%–48.4% (oleic acid) among tree samples. Significant tree-to-tree variation in the mass of the fruits, number of seeds in each fruit, and chemical constituents of the seed oil were observed [42]. On one hand, these results allowed the researchers to identify a potential breeding population (trees that produced seeds with desired characteristics), but on the other hand, the high degree of variation in both physical and chemical properties of the fruits and seeds of the wild *A. floribunda* trees highlights some of the inherent problems encountered when attempting to establish a steady supply chain of high-quality *Allanblackia* oil from seeds collected in the wild.

The optimum conditions for the extraction of seed oil from several species of *Allanblackia* have yet to be determined. One study, however, has been conducted with *A. floribunda* seeds to determine the optimum extraction conditions and assess both the quality and stability of the oil obtained from crude pressing methods and from solvent extraction. In this study, the seed samples were milled under different temperature regimes and moisture levels, and the oil was either expressed by use of a manual screw press or a Soxhlet apparatus (solvent: petroleum ether). The oil yield obtained from solvent extraction was higher than from the manual expression (67.6% *vs.* 48.6%, respectively), but the quality parameters measured such as the melting point, acid value, ester value, iodine value, peroxide value, refractive index, specific gravity, and saponification value showed no significant differences between the oils. The pressed oil, however, due to significant differences in the peroxide value and free fatty acid content, was more stable when stored in plastic containers as compared to the oil extracted with solvent [43].

While the quality parameters of the expressed or extracted oil are of particular importance, the toxicological and environmental aspects of seed oil extraction also require consideration. The use of hot water as an extraction solvent, due to its low cost, toxicity, and environmental impact, was investigated as a method of extracting oil from *A. floribunda* seeds. An extraction yield of 42.2% oil (*w*/*w*) from the seeds and extraction efficiency of 58.6% was reported by Alenyorege *et al.* [44], indicating that this method could be applied to meet commercial, industrial, and domestic demands.

#### 2.4.2. *Allanblackia parviflora* Seed Oil

The seed oil from *A. parviflora* has been shown to be quite similar to that from *A. floribunda*. An early study of the fatty acid components of *A. parviflora* revealed them to be primarily stearic acid (51.6%) and oleic acid (43.9%), with minor amounts of myristic acid (1.8%), palmitic acid (2.5%), and eicosanoic acid (0.2%). The major triglyceride component were determined as 2-oleostearin (60.1%), 1-stearo-diolein (26.9%) and 2-oleopalmitostearin (6.9%) [39]. A study conducted on seeds of *A. parviflora* growing in Ghana, from which oil was extracted by either the use of a screw press or Soxhlet extraction (petroleum ether), yielded an average of 68% oil (*w*/*w*). The fatty acid composition of the seed oil was determined by gas chromatography as 2.9% palmitic acid, 52.3% stearic acid, and 44.8% oleic acid. Secondary metabolites belonging to the classes of carotenoids, terpenes, saponins, or tannins were not detected in the oil in this study. Nutritional analyses indicated that the seeds contained 4.3% protein, 2.0% ash, 5.7% crude fiber, and 17.1% carbohydrates [16]. These values were generally lower than those reported for shea kernels and cocoa beans, but the energetic value of *Allanblackia* seeds was 2863.44 kJ/100 g (exceeding both that of shea kernels and cocoa beans), supporting the traditional consumption of these seeds as a high-energy snack in some parts of Africa [11].

#### 2.4.3. *Allanblackia stuhlmannii* Seed Oil

A triglyceride derived from three molecules of stearic acid (2-oleostearin) was first isolated by crystallization from *A. stuhlmannii* seed oil in 1896, in amounts that were even remarked upon as being impressive at the time [45]. The seed oil of *A. stuhlmannii* displays some similarities to that of *A. floribunda* and *A. parviflora*. The edible oil is solid at room temperature (melting point (mp) = 34 °C) and, in Tanzania, is referred to by the common names “*Allanblackia* fat” or “kanye butter” [46]. Due to its physical properties and neutral taste, it is used in cooking. The seeds have been reported contain *ca.* 50% oil (*w*/*w*), the fatty acid composition of which consists mainly of stearic acid (45%–58%) and oleic acid (40%–51%) [12].

#### 2.4.4. Other *Allanblackia* Seed Oils

The seed oils of two species of *Allanblackia* occurring in Cameroon, *A. gabonensis* and *A. stanerana*, have been examined, whereby the oil was extracted through maceration of the ground seeds with hexane. The seeds of *A. gabonensis* were demonstrated to yield 68.2% oil (*w*/*w*), which contained 5.35% water and 60.6% saturated, 37.6% monounsaturated, and 0.8% polyunsaturated fatty acids, of which the C18:0 and C18:1 types dominated. The seeds of *A. stanerana*, meanwhile, yielded 69.9% oil (*w*/*w*), which contained 22.0% water and 70.9% saturated, 28.2% monounsaturated, and 0.8% polyunsaturated fatty acids, and again, the C18:0 and C18:1 types dominated. The quality parameters, namely the acid index, iodine index, and refractive index values, were similar for these two oils, although the former two parameters were higher for *A. gabonensis* and the latter parameter, higher for *A. stanerana*. In both seed oils, the primary saturated fatty acid was stearic acid (60.1% and 69.6%, respectively), while the major monounsaturated fatty acid was oleic acid (37.4% and 28.1%, respectively [47]. The seed oils of *A. kimbiliensis*, *A. kisonghi*, *A. marienii* and *A. ulugurensis* have not yet been subjected to physicochemical or phytochemical analyses to this author’s knowledge.

### 2.5. Phytochemistry and Medicinal Use of Allanblackia Seeds

In western Africa, decoctions of the leaves and bark of *A. floribunda* are used to treat toothache, dysentery, and hypertension and the crushed plant material or extracts is applied topically as an analgesic. Specific medicinal applications for the seeds, however, in the traditional medicinal systems found in this region, and no bioactive secondary metabolites have yet been isolated from the plant [48]. In Tanzania, various parts of *A. stuhlmannii* are used in the traditional medicine; the leaves are chewed to treat coughs, a leaf tea is drunk to treat chest pain, and extracts of the roots, bark, and leaves are taken to treat impotence. Interestingly, the heated oil extracted from the seeds is used as a liniment to treat rheumatism, rubbed into sore joints, or dabbed on wounds and rashes [49]. The Hehe people, native to south-central Tanzania, have been reported to combine the seed oil with crushed seeds of *Psorospermum febrifugum* Spach (Hypericaceae) and rub the mixture into the soles of the feet to heal deep cracks [46]. Although no phytochemical investigations of *A. stuhlmanniii* seeds have yet been conducted, the seeds of *Psorospermum febrifugum* have been shown to contain numerous bioactive xanthones [50].

Interestingly, a xanthone derivative (allanxanthone E), several xanthones (1,7-dihydroxy-3-methoxy-2-(3-methylbut-2-enyl)xanthone; α-mangostin; garciniafuran; allanxanthone C; and 1,6-dihydroxy-2,4-diprenylxanthone) and two triterpenes (friedelin and lupeol) have been isolated from the seeds of *Allanblackia gabonensis* (cited under the synonym *A. monticola* Mildbr. ex Engl.), which is widely distributed in western Cameroon. This species is used in the local traditional medicine to treat diarrhea, fever, pain, respiratory infections, and toothache. α-mangostin was shown to possess apoptotic and antiproliferative activity against ESKOL cells derived from a hairy cell leukemia patient and leukemia cells freshly isolated from B-CLL patients when tested in concentrations as low as 2.4 μM. Allanxanthone E, 1,7-dihydroxy-3-methoxy-2-(3-methylbut-2-enyl)xanthone, and α-mangostin triggered apoptosis in B-CLL leukemia cells in a dose-dependent manner, whereby the latter compound was the most potent [51].

These results are significant when considering the inclusion of *Allanblackia* oils in functional foods, especially unrefined oils and particularly those that have been extracted using solvents with intermediate polarity. As shown for the case of the benzophenone derivatives, guttiferone E and F (Section 2.2), bioactive secondary metabolites can be co-extracted along with primary metabolites such as triacylglycerols and fatty acids from the seeds of *Allanblackia*. When ingested, these phytochemicals have the potential to act as pharmacological agents, and could prevent the development of or be used to treat chronic disease. This said, very few studies to date have carefully examined either manually expressed or solvent extracted *Allanblackia* seed oils for the presence, identity and/or quantity of potentially bioactive secondary metabolites, and this is both an interesting and important direction for future research.

## 3. Conclusions

Species of the genus *Allanblackia* (Clusiaceae) are currently the focus of a high degree of attention, due to interest in the consumption and commercial exploitation of the seed oils. The steadily increasing demand for alternative, plant-based oils and fats and the favorable physicochemical characteristics of *Allanblackia* seed oils (with up to 95% combined stearic and oleic acid) have led to its inclusion as an ingredient in functional food products, including vegetable-based dairy products, ice cream, and spreads. While domestication projects are underway to establish the sustainable cultivation of several *Allanblackia* species in tropical Africa, the steadily growing demand for the seed oil has resulted in increased harvesting pressures on wild populations of *Allanblackia* species and, in turn, increased the risk of misidentification and/or adulteration. Proper botanical identification of the species used is important, since only authenticated oil can be deemed safe for human consumption when included in functional food products.

In terms of its nutritional value, *Allanblackia* oil can be combined with other oils or fats to achieve specific physical properties. The high stearic acid content (ranging, according to the species, between 44%–66%) and comparatively low palmitic acid content of the oil is relevant because stearic acid has not been demonstrated to alter the plasma levels of total low density lipoprotein (LDL, or “bad” cholesterol) and high density lipoprotein (HDL, or “good” cholesterol), unlike palmitic acid. Functional food products containing *Allanblackia* oil, therefore, could be considered to have health advantages over products containing oils with higher levels of lauric, myristic, and/or palmitic acids, the ingestion of which has been shown to lead to increases in blood cholesterol levels. Such considerations may be particularly important for individuals at greater risk of cardiovascular disease or who have hypercholesterolemia.

Information available about the chemical constituency of *Allanblackia* seed oils, the roles of the component phytochemicals in preventive strategies (e.g., associated with increased intake as part of a healthy diet), or the role of these compounds as pharmacological agents used in the treatment of chronic disease was examined in this review. The phytochemistry of the seed oils of nearly all *Allanblackia* species is poorly known, particularly with regard to the presence and/or content of lipophilic secondary metabolites. Unrefined seed oils from *Allanblackia* have been shown to contain bioactive secondary metabolites (*i.e.*, benzophenone derivatives, xanthones), which may have the potential to act as pharmacological agents when ingested. Phytochemical investigations of *Allanblackia* seed oils for the presence, identity and/or quantity of potentially bioactive secondary metabolites, and pharmacological testing of isolated compounds represent important directions for future research.
